# Tradeoff between Stability and Maneuverability during Whole-Body Movements

**DOI:** 10.1371/journal.pone.0021815

**Published:** 2011-07-14

**Authors:** Helen J. Huang, Alaa A. Ahmed

**Affiliations:** Department of Integrative Physiology, University of Colorado at Boulder, Boulder, Colorado, United States of America; Universidad Europea de Madrid, Spain

## Abstract

**Background:**

Understanding how stability and/or maneuverability affects motor control strategies can provide insight on moving about safely in an unpredictable world. Stability in human movement has been well-studied while maneuverability has not. Further, a tradeoff between stability and maneuverability during movement seems apparent, yet has not been quantified. We proposed that greater maneuverability, the ability to rapidly and purposefully change movement direction and speed, is beneficial in uncertain environments. We also hypothesized that gaining maneuverability comes at the expense of stability and perhaps also corresponds with decreased muscle coactivation.

**Materials and Methods:**

We used a goal-directed forward lean movement task that integrated both stability and maneuverability. Subjects (n = 11) used their center of pressure to control a cursor on a computer monitor to reach a target. We added task uncertainty by shifting the target anterior-posterior position mid-movement. We used a balance board with a narrow beam that reduced the base of support in the medio-lateral direction and defined stability as the probability that subjects could keep the balance board level during the task.

**Results:**

During the uncertainty condition, subjects were able to change direction of their anterior-posterior center of pressure more rapidly, indicating that subjects were more maneuverable. Furthermore, medio-lateral center of pressure excursions also approached the edges of the beam and reduced stability margins, implying that subjects were less stable (i.e. less able to keep the board level). On the narrow beam board, subjects increased muscle coactivation of lateral muscle pairs and had greater muscle activity in the left leg. However, there were no statistically significant differences in muscle activity amplitudes or coactivation with uncertainty.

**Conclusions/Significance:**

These results demonstrate that there is a tradeoff between stability and maneuverability during a goal-directed whole-body movement. Tasks with added uncertainty could help individuals learn to be more maneuverable yet sufficiently stable.

## Introduction

A stability-maneuverability tradeoff during locomotion [1] and posture [2], [3] seems apparent yet there are no experimental studies that quantify this tradeoff in humans. One difficulty of studying stability and maneuverability is that there is not a consensus on the precise definitions and metrics of stability and maneuverability. Stability has a range of definitions and is difficult to define [2], [4]. In general, stability relates to remaining in a particular state or maintaining a particular set of dynamics. There are numerous studies that examine stability in human movement [5], [6], [7], [8], [9], [10], [11], [12], [13]. The definition of maneuverability is less debatable, and generally relates to turning ability, which involves purposeful changes in movement direction and/or speed. Unlike stability, fewer studies have examined maneuverability in human movement [1], [2].

Regardless of the precise definitions of stability and maneuverability, understanding how stability and/or maneuverability affects motor control strategies can provide insight on moving about safely in an unpredictable world. An inability to adapt to various demands of stability and maneuverability may hinder performance of daily tasks such as gait initiation, opening a door, or reaching for a plate in a cupboard. During conditions of instability, people use muscle coactivation to increase joint, endpoint, and/or limb stiffness [5], [14], [15], [16]. This increase in stiffness attenuates movement deflection for a given force perturbation and is often assumed to enhance stability [17]. However, overly stiff and overdamped joints can also impair corrective responses [17], [18], possibly hindering the ability to make a maneuver. Additionally, individuals who over-emphasize the need for stability such as older adults, may self-restrict their movement capacity [19], [20], [21]. For example, older adults tend to have high levels of muscle coactivation [15], [22], [23] and also tend to move and walk more slowly [24], [25], suggesting they may be less maneuverable.

**Figure 1:**
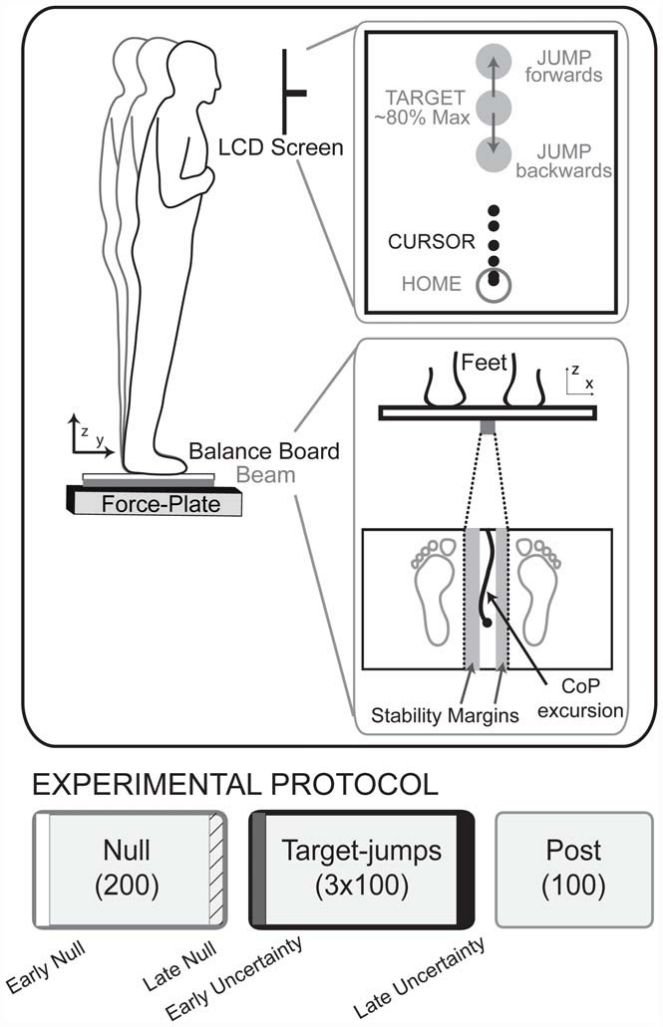
Experimental setup and protocol. Subjects stood on a balance board with a narrow beam of support and performed forward leans to move a cursor via their center of pressure to a target presented on a LCD screen. To add uncertainty, targets may jump forwards, backwards, or remain at the original target position. A top down view of the balance board and feet illustrates that stability margins represent the difference between the width of the beam and the range of medio-lateral center of pressure (CoP) excursion. The experimental protocol consisted of 200 null trials, 300 target-jump trials, and 100 post trials. During the target-jump block, each of the three possible target-jump distances were presented 100 times in a randomized order. Key time points for comparison were Late Null (diagonal hatch), Early Uncertainty (gray fill), and Late Uncertainty (black fill).

The purpose of this study was to determine if a tradeoff between stability and maneuverability exists during whole-body movements. To test this, we used a forward lean movement task that integrated both stability and maneuverability (Figure 1). Subjects used their center of pressure to control a cursor on a computer monitor to reach a target. We used a balance board with a narrow beam that reduced the width of the base of support in the medio-lateral direction, challenging frontal plane stability and likely necessitating an increase in muscle coactivation. By increasing the need for coactivation, we could determine how muscle activity and coactivation change in relation to the tradeoff. We defined stability as the probability that subjects could keep the balance board level during the forward lean movement. We measured the stability margin, the distance from the edge of the beam that subjects maintained during the movement task, as our stability metric (Figure 1). In accordance with our operational definition of stability, we predicted that smaller stability margins likely corresponded with a greater chance of tilting the board, implying less stability. We added task uncertainty by using anterior-posterior direction target-jumps that shifted the target position mid-movement to increase maneuverability demands (Figure 1). There were three types of target-jumps: a forward shift (jump-f), backward shift (jump-b), or no shift (jump-0). The backward target-jump specifically probed maneuverability because it required a reversal of movement direction. Null trials did not involve a target-jump and the target remained at the original target location.

We first hypothesized that the uncertainty condition (target-jumps) would encourage subjects to adopt more maneuverable motor control strategies. This would indicate that maneuverable motor control strategies are beneficial for adapting to unpredictable conditions. We then hypothesized that to gain maneuverability, subjects would sacrifice stability. This would indicate that there is a stability-maneuverability tradeoff in whole-body movements. Lastly, we hypothesized that maneuverable strategies corresponded with decreased muscle coactivation. Because individuals tend to increase muscle coactivation to gain a sense of greater stability, a logical deduction was that individuals would exhibit the contrapositive relationship, that is less stability (i.e. greater maneuverability from the tradeoff) resulted from decreased muscle coactivation.

## Results

Subjects adopted more maneuverable but less stable center of pressure control strategies during a condition of increased uncertainty.

### Maneuverability metric: response time

We quantified the time subjects needed to reverse their anterior-posterior center of pressure excursion in response to a backwards target-jump (jump-b). The backward target-jump required a maneuver, in this case a purposeful reversal of direction, that was easily identifiable, compared to the forward target-jump. We compared the response times of early and late backwards target-jump. Early corresponded to the first 15 successful trials and late was the last 15 successful trials. Successful trials were trials where the board did not tilt and subjects reached the target in less than 2.5 seconds. Faster response times reflect a more maneuverable strategy.

**Figure 2:**
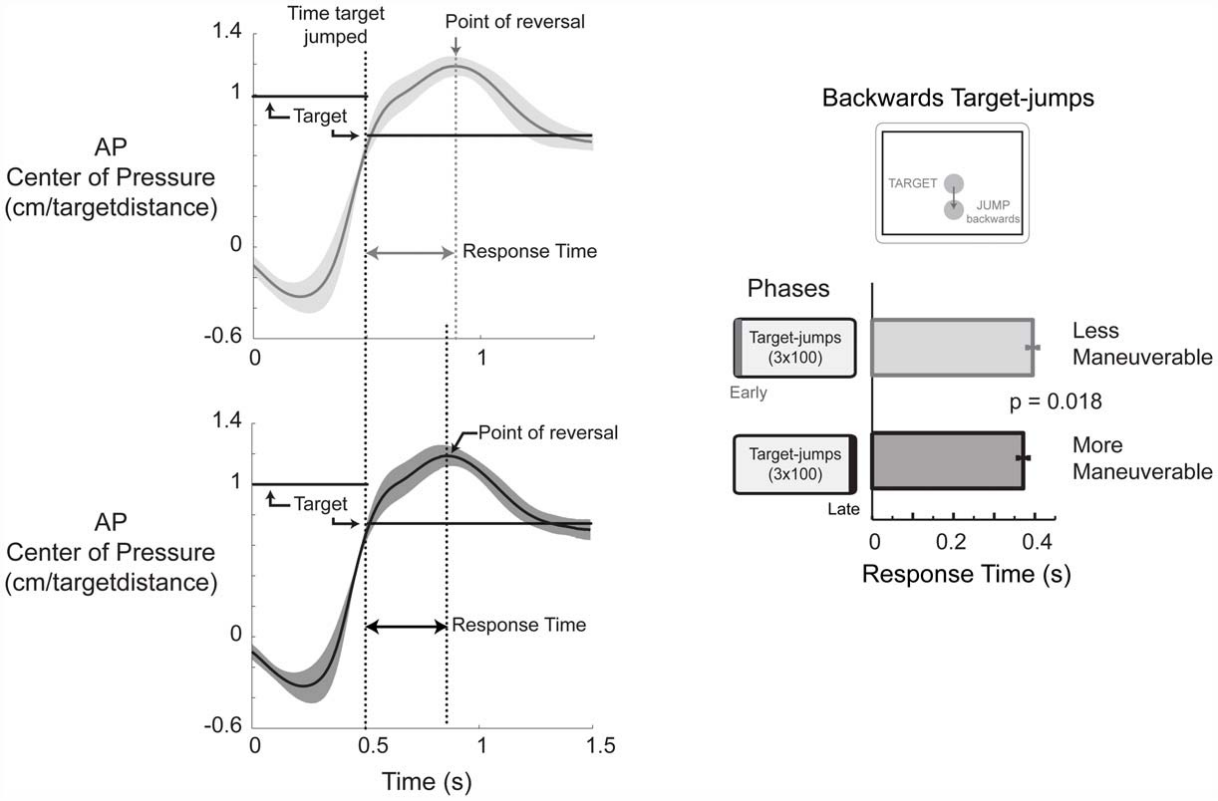
Uncertainty promotes maneuverability. Group averaged (n  = 11) anterior-posterior (AP) center of pressure time series profiles for early (light gray) and late (dark gray) backwards target-jumps. Solid lines are group means and shaded areas are±s.d. Subjects had shorter response times at late versus early target-jump. The maneuverability metric was the response time required for subjects to reverse center of pressure direction in response to a mid-movement backwards target-jump. Bars are the group means±s.e.m. response times for each time point.

Subjects were able to respond significantly quicker, reversing directions in 0.371±0.045 seconds (mean±sd) by late backwards target-jump compared to 0.395±0.046 seconds during early backwards target-jump (Figure 2, *p* = 0.018). The response time data set had a normal distribution, Shapiro-Wilk p = 0.82.

### Stability metric: stability margin

We quantified stability margin as the difference between the beam width and the range of medio-lateral center of pressure excursion during each trial. The stability margin thus quantified the buffer between the subject's center of pressure excursion and the edge of the beam. We compared the stability margins of late null to late jump-0. Late corresponded to the last 15 successful trials. The target distance for these target-jump trials was the same as the target distance during the null trials. A change in stability margin would reflect a change in feed-forward strategy as a result of the added uncertainty during the target-jump condition. A decrease in stability margin would imply a less stable strategy. We also calculated the percentage of successful trials out of all attempts within a bin of stability margin across the width of the narrow beam. This calculation checked that stability margin correlated with percent success and was consistent with our operational definition of stability.

**Figure 3:**
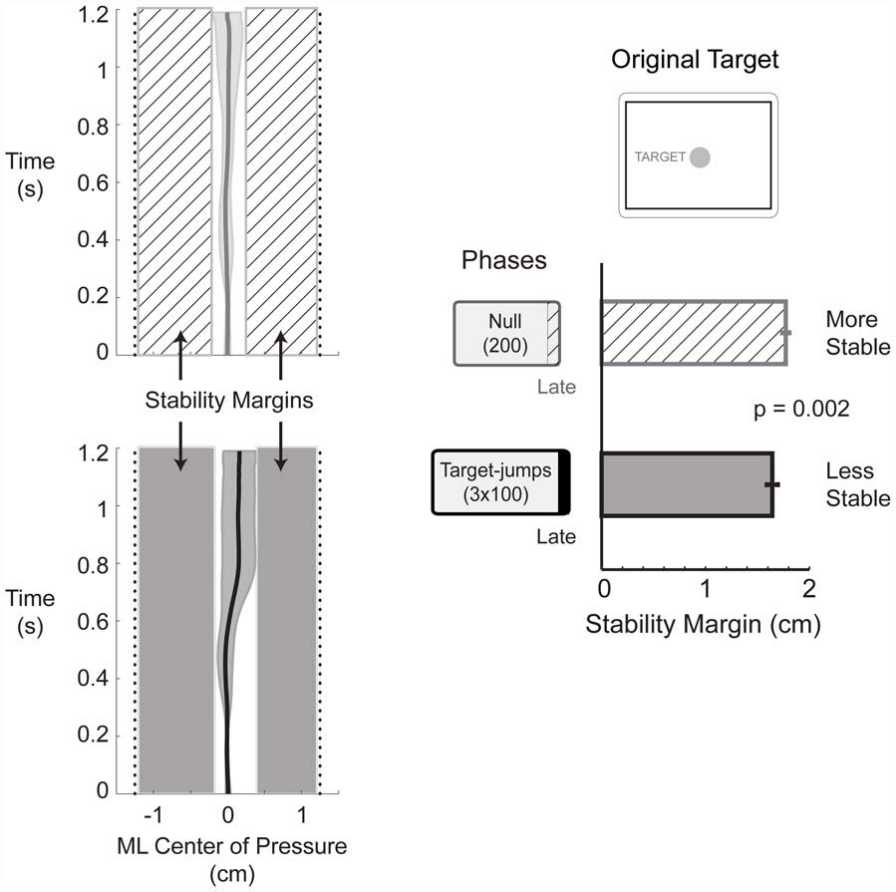
Maneuverability comes at the expense of stability. Group averaged (n  = 11) medio-lateral (ML) center of pressure time series profiles for late null (light gray) and late target-jump (dark gray). These conditions compared trials to the same target distance. Solid lines are group means and shaded areas are±s.d. The stability metric was the width of the stability margins which were larger for late null (diagonal hatch) compared to late target-jump (dark gray). Bars are the group means±s.e.m. of the stability margin for each time point.

Subjects reduced their stability margins for a movement to the same target distance from 1.77±0.11 cm during late null to 1.64±0.18 cm during the uncertainty (target-jump) condition (Figure 3, *p* = 0.002). The stability margin data set had a normal distribution, Shapiro-Wilk p = 0.70. This decrease in stability margin emerged when stability was challenged on the narrow beam balance board, but not during the practice set on the wide beam balance board. On the wide beam balance board, stability margins became larger, progressing from 1.53±0.36 at late null to 1.54±0.28 at late jump-0 to 1.63±0.26 cm at late post. For completeness, Table 1 contains center of pressure measures for all phases of the experiment, including the practice set on the wide beam balance board.

**Table 1:** Center of pressure (CoP) measures (mean±s.d.) for all phases of the experiment.

		NULL		UNCERTAINTY/TARGET-JUMPS						POST	
				Jump-b		Jump-0		Jump-f			
		*Early*	*Late*	*Early*	*Late*	*Early*	*Late*	*Early*	*Late*	*Early*	*Late*
**Stability Margin (cm)**	***WIDE (practice)***	1.74±0.21	1.53±0.36	1.42 ±0.33	1.45±0.25	1.43±0.38	1.54±0.28	1.33±0.40	1.44±0.31	1.53 ±0.28	1.63±0.26
	***NARROW***	1.61±0.17	***1.77*** **±** ***0.11***	1.54±0.18	1.57±0.17	1.62±0.14	***1.64*** **±** ***0.18*** *	1.53±0.15	1.55±0.16	1.66±0.21	1.73±0.20
**Range of ML CoP excursion (cm)**	***WIDE(practice)***	0.80±0.21	1.01±0.36	1.13±0.33	1.09±0.25	1.11±0.38	1.00±0.28	1.21±0.40	1.10±0.31	1.01±0.28	0.91±0.26
	***NARROW***	0.93±0.17	0.77±0.11	1.00±0.18	0.97±0.17	0.92±0.14	0.90±0.18	1.01±0.15	0.99±0.16	0.88±0.21	0.81±0.20
**Range of AP CoP excursion (% null target distance)** □	***WIDE (practice)***	1.41±0.14	1.42±0.14	1.59±0.17	1.62±0.16	1.56±0.17	1.57±0.17	1.72±0.13	1.73±0.14	1.50±0.14	1.46±0.14
	***NARROW***	1.32±0.11	1.43±0.07	1.57±0.15	1.56±0.15	1.53±0.13	1.53±0.13	1.73±0.12	1.74±0.11	1.50±0.10	1.49±0.10
**Response time (s)**	***WIDE (practice)***	na	na	0.41±0.05	0.38±0.04	na	na	na	na	na	na
	***NARROW***	na	na	***0.40*** **±** ***0.05***	***0.37*** **±** ***0.04*** *	na	na	na	na	na	na

CoP excursion ranges in the anterior-posterior (AP) and medio-lateral (ML) directions for the early and late phases during the null, jump-0, jump-b, jump-f, and post blocks. Early consisted of the first 15 successful trials and late were the last 15 successful trials.

□Subjects had different target distances.

na = not applicable. Response times only calculated when subjects were forced to make a maneuver during the backwards target-jump.

Bold text highlights planned comparisons.

*Significantly different, p<0.05.

**Figure 4:**
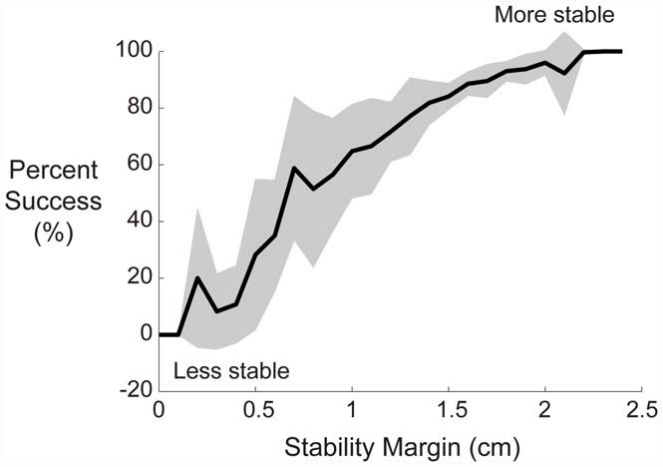
The percent of success and stability. Percent of successful trials in 0.1 cm bins of stability margin across the width of the beam of support. Thick line is the mean and the shaded area is±s.d. Larger stability margins corresponded with higher probabilities of success and implied increased stability. Smaller stability margins corresponded with lower probabilities of success and implied decreased stability. These data support the definition of stability as the probability of keeping the board level and of stability margin as a metric of stability.

Further, smaller stability margins corresponded with decreased stability, in that subjects were less successful at the movement task (Figure 4). This demonstrated that smaller stability margins corresponded with low success probabilities and thus reduced stability. Larger stability margins corresponded with higher success probabilities and greater stability. Similarly, other postural control studies have also interpreted a decrease of stability margin to be less stable [9], [13].

### Stability-maneuverability tradeoff

**Figure 5:**
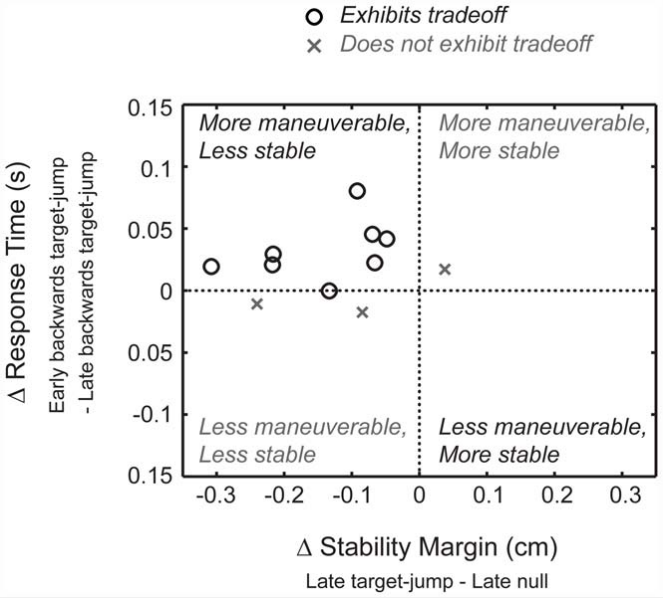
Stability-maneuverability tradeoff for individual subjects. Change in response times (early and late jump-b) versus change in stability margins (late null and late jump-0) for each individual subject. A positive change in response time indicated a faster response time by late target-jump. A positive change in stability margin indicated larger stability margins by late target-jump. Four quadrants characterize changes in movement strategy to be 1) more maneuverable/more stable, 2) more maneuverable/less stable, 3) less maneuverable/less stable, and 4) less maneuverable/more stable. Circles represent subjects (n = 8) who exhibited a stability-maneuverability tradeoff whereas X's represent subjects (n = 3) who did not exhibit the tradeoff.

Eight out of eleven subjects exhibited a stability-maneuverability tradeoff (Figure 5 o's). These subjects changed their center of pressure control strategy to be more maneuverable/less stable. The other three subjects exhibited a change in center of pressure control strategy that indicated a shift toward being more maneuverable/more stable or less maneuverable/less stable (Figure 5 x's).

### Muscle activation and coactivation

We recorded muscle activity from the tibialis anterior (TA), soleus (SO), medial gastrocnemius (MG), lateral gastrocnemius (LG), peroneus longus (PL), the long head of the biceps femoris (BF), rectus femoris (RF), and vastus lateralis (VL) on each lower limb. We quantified the root-mean-square (RMS) of the 100 ms prior to the cursor leaving the home circle and then averaged the RMS EMG of this 100 ms bin for the last 15 successful null trials during the practice set on the wide balance board as the normalization value. With this normalization value, we could more easily interpret changes in EMG during the trial and across trials, phases, and beams. We calculated the RMS EMG from the 100 ms prior to the cursor leaving the home circle until the cursor settled in the target to quantify muscle activity amplitude for each trial. We averaged the RMS EMG of the trials during late null, late jump-0, early jump-b, and late jump-b. We then compared the average RMS EMG for late null to late jump-0, and for early jump-b to late jump-b phases. A decrease in RMS EMG between these phases would suggest that less muscle activity corresponded with a more maneuverable and less stable strategy.

**Figure 6:**
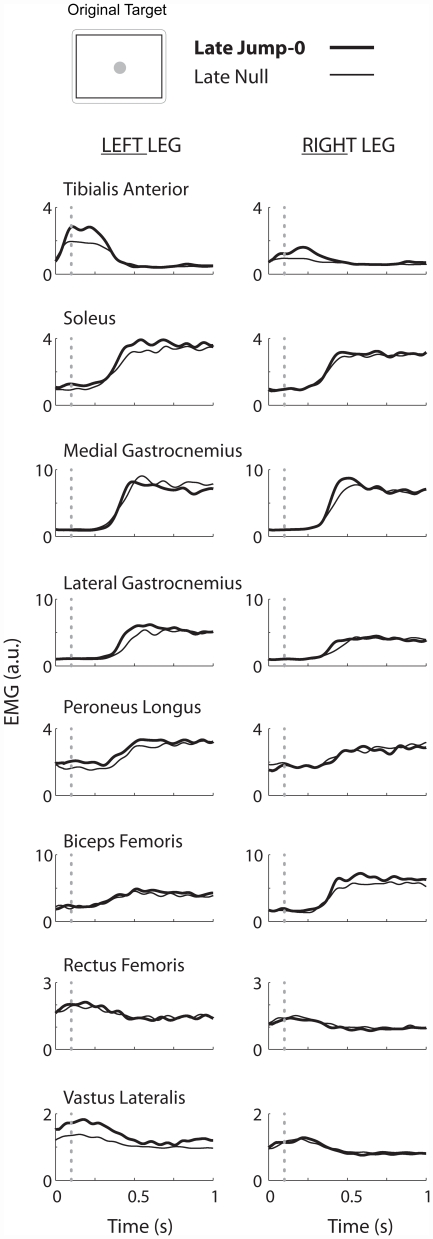
Group averaged muscle activity linear envelopes for late null and late jump-0. Linear envelopes were similar between late null (thin line) and late jump-0 (thick line). The dashed vertical line indicates when the cursor moved out of the home circle.

**Figure 7:**
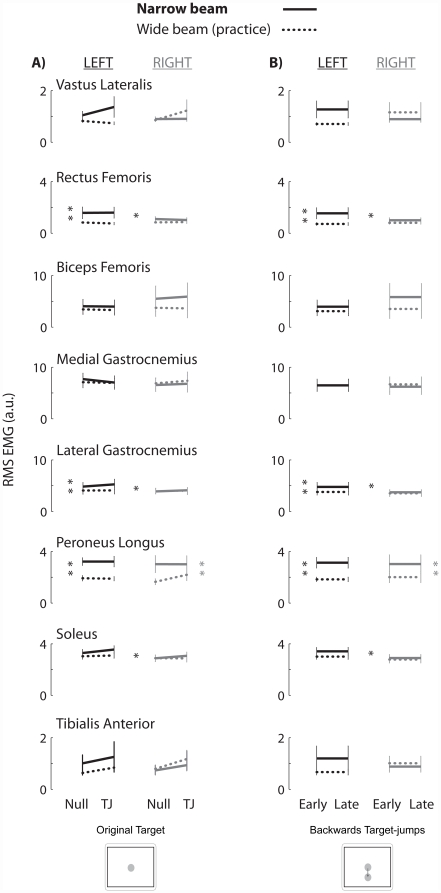
Group averaged RMS EMG amplitudes for all 16 lower limb muscles. Thick lines are the narrow beam data while dotted lines are the wide beam data. Left muscles are black and right muscles are gray. Single asterisks indicate a significant increase in left muscle activity compared to the right muscle on the narrow board. Black double asterisks indicate a significant increase in muscle activity in the left muscle on the narrow beam compared to the wide beam board, while gray double asterisks indicate a significant increase in muscle activity in the right muscle. Error bars are standard error of the mean.

No statistically significant differences in muscle activity were found between late null and late jump-0 or between early and late jump-b phases, even though differences in stability margin and response time were statistically significant. As expected, the tibialis anterior muscles were active during the initial backwards center of pressure movement. When the center of pressure moved forwards, the plantarflexors (medial gastrocnemius, lateral gastrocnemius, and soleus) were active. The tibialis anterior burst did not overlap much with the plantarflexor activity. Group averaged linear envelopes were similar between late null and jump-0 (Figure 6). Group averaged RMS EMG amplitudes between late null and late jump-0 within a beam (ANOVA *p*'s>0.58, Figure 7A) and between early and late jump-b (ANOVA *p*'s>0.74, Figure 7B) were not statistically significant for any muscle. Muscle activity amplitudes on the narrow beam were significantly greater than on the wide beam for the left rectus femoris, left lateral gastrocnemius, and the left and right peroneus longus muscles (THSD *p's*<0.05, Figure 7). There was also a significant asymmetrical increase in muscle activity of the rectus femoris, lateral gastrocnemius, and soleus muscles in the left leg compared to the right leg on the narrow beam (THSD *p's*<0.05, Figure 7).

**Figure 8:**
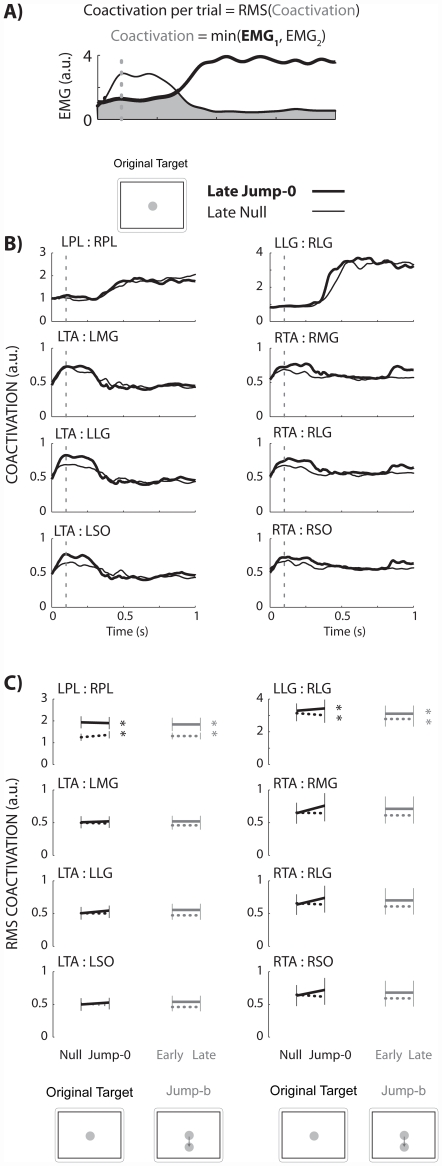
Schematic of coactivation definition, coactivation linear envelopes, and group averaged RMS coactivation amplitudes. A) Coactivation was the minimum value of EMG1 and EMG2. B) Coactivation linear envelopes between late null (thin line) and late jump-0 (thick line) were similar. The dashed vertical line indicates when the cursor moved out of the home circle. C) Thick lines are the narrow beam data while dotted lines are the wide beam data. Late null and late jump-0 are in black. Early and late jump-b are gray. Black double asterisks indicate a significant increase in coactivation in the lateral muscle pairs on the narrow beam compared to the wide beam board during late null to late jump-0. Similarly, gray double asterisks indicate a significant increase in coactivation on the narrow beam during early to late jump-b. Error bars are standard error of the mean.

We quantified coactivation of two lateral muscle pairs: 1) left and right peroneus longus (LPL:RPL) and 2) left and right lateral gastrocnemius muscles (LLG:RLG). We also quantified coactivation of three ankle muscle pairs on the left (L) and right (R) lower limbs: 1) tibialis anterior and soleus (LTA:LSO, RTA:RSO), 2) tibialis anterior and medial gastrocnemius (LTA:LMG, RTA:RMG), and 3) tibialis anterior and lateral gastrocnemius muscles (LTA:LLG, RTA:RLG). For each time point in a trial, the minimum normalized EMG activity level of the muscle pair was determined, yielding a coactivation profile for the trial (Figure 8A). This coactivation profile represented the “wasted contraction” [26], [27]. We then calculated the RMS of the coactivation profile to get a coactivation amplitude per trial. We compared the coactivation amplitudes of late null to late jump-0. Again, late corresponded to the last 15 successful trials. A decrease in coactivation by late target-jump would indicate that a more maneuverable, less stable strategy uses less coactivation.

There were no statistically significant differences in muscle coactivation between late null and late jump-0 or between early and late jump-b phases. Group averaged linear envelopes of coactivation were similar between late null and jump-0 (Figure 8B). Group averaged RMS coactivation amplitudes within a beam between late null and late jump-0 (ANOVA *p*'s>0.29) and between early and late jump-b (ANOVA *p*'s>0.29) were not statistically significant (Figure 8). Coactivation amplitudes on the narrow beam were significantly greater than the wide beam for left-right peroneus longus pair and for the left-right lateral gastrocnemius pair (THSD *p*'s<0.05, Figure 8C).

## Discussion

Subjects demonstrated a stability-maneuverability tradeoff during a condition with increased uncertainty. Subjects adopted a motor control strategy that enabled them to make a maneuver more quickly, thus demonstrating greater maneuverability in response to increased uncertainty. Increased maneuverability, however, also came at the expense of stability. To achieve greater maneuverability, subjects adopted a motor control strategy that reduced their stability margins, moving their center of pressure closer to the edges of the beam. There were no statistically significant differences in muscle activity or coactivation between late null and late jump-0 and between early and late jump-b, even though there were significant differences in stability margin and response times.

The stability-maneuverability tradeoff demonstrated in this experiment was not the consequence of definitions or behaviors being inversely related. In this experimental setup, subjects could have completed the movement task successfully, although more slowly, without sacrificing stability. We chose to manipulate stability in the frontal plane because 1) medio-lateral postural instability may contribute to falls [8], [28] and 2) active balance control during walking occurs in the medio-lateral direction [29]. Because the stability manipulation was in the medio-lateral direction, stability demands were orthogonal to the movement task which was in the anterior-posterior direction. This orthogonality permitted the independent control of anterior-posterior movements and of maintaining medio-lateral stability. Thus, changes in anterior-posterior movements could be made without altering stability margins and medio-lateral control. Yet, our data revealed that despite the orthogonality of the movement task and reduced base of support, stability and maneuverability control opportunistically interacted and demonstrated a tradeoff. The orthogonality between stability and maneuverability highlights the benefit and significance of the tradeoff. We would expect that if stability and maneuverability acted along the same axis, then the tradeoff would be even more prominent, however this experiment did not test for a tradeoff acting along the same axis.

Our data also revealed that the stability-maneuverability tradeoff was not always exhibited. Three subjects did not demonstrate the tradeoff on the narrow beam. These subjects may have had an alternative strategy for dealing with the uncertainty, by responding less quickly or by preferring stability. Another explanation is that the orthogonality of our experimental setup diminishes the need for the tradeoff. Interestingly, when stability was not challenged (i.e. wide beam balance board), subjects had faster response times, yet used similar stability margins at late target-jump compared to late null (Table 1). The stability-maneuverability tradeoff emerged on the narrow beam balance board when stability was not guaranteed or when stability was limited. Overall, the data suggest that the body is opportunistic, exploiting the stability-maneuverability tradeoff for the benefit of achieving task goals. Similarly, in a goal-directed arm reaching task, humans also exhibited opportunistic control by exploiting a stability-accuracy tradeoff [18].

The stability-maneuverability tradeoff observed was unlikely to result from learning or from having longer movement paths. Subjects were given substantial practice, performing the entire protocol on a wide beam balance board. These practice trials allowed subjects to focus on learning a successful strategy for reaching the target. With practice, humans tend to increase stability margins when learning a dynamic task [9]. On the wide beam balance board, our subjects also learned to have narrower medio-lateral center of pressure excursion paths and larger stability margins (Table 1, late null to late post). Thus, if learning was the dominant factor of stability control, then subjects would also have narrower center of pressure excursions and wider stability margins at late target-jump on the narrow beam balance board. Yet, subjects used wider medio-lateral center of pressure excursions and smaller stability margins which was not consistent with the effect of learning. Furthermore, increases in medio-lateral center of pressure excursions were not necessarily the consequence of making larger anterior-posterior center of pressure excursions. The range of anterior-posterior center of pressure excursions for late null on the wide and narrow beam balance boards were not significantly different, 1.42±0.14 cm and 1.43±0.07 cm respectively; however, the range of medio-lateral center of pressure excursions were significantly different, 1.01±0.36 cm and 0.77±0.11 cm, respectively. On the other hand, the range of anterior-posterior center of pressure excursions for late backwards and late forwards target-jumps on the narrow beam were significantly different, 1.56±0.15 cm and 1.74±0.11 cm respectively; however, the range of medio-lateral center of pressure excursions were not significantly different, 0.97±0.17 cm and 0.99±0.16 cm, respectively. Thus, smaller stability margins and wider medio-lateral excursions were not necessarily the consequence of making larger anterior-posterior movements.

We did not find significant differences in muscle activity and coactivation between phases, despite significant changes in response time and stability margins. There were statistically significant increases in the RMS coactivation for the left-right peroneus longus pair and left-right lateral gastrocnemius pair (Figure 8C) on the narrow beam board compared to the wide beam board. One reason we used a narrow beam board was to necessitate an increase in muscle coactivation so that subjects could possibly decrease muscle coactivity during the experiment. The coactivation results highlight that subjects increased coactivation in a direction specific manner and only increased coactivation of lateral muscle pairs on the narrow beam board which challenged medio-lateral stability. Because the movement task was in the anterior-posterior direction and there were no significant changes in coactivation among the tibialis anterior and plantarflexors muscle pairs, we hypothesized that there may be changes in individual muscles. On the narrow beam board, there were significant increases in RMS EMG for the left rectus femoris, left lateral gastrocnemius, and left and right peroneus longus muscles (Figure 7). This asymmetrical increase of muscle activity between the left and right legs could possibly explain the decreased stability margins and increased maneuverability on the narrow beam board; however, there were no statistically significant differences in RMS EMG in any muscle between late null and late jump-0 or between early and late jump-b.

One possible explanation for the non-significant coactivation results between phases was that the center of pressure movements and velocities were not large enough to produce sufficiently large changes in muscle activity. An additional difficulty was that numerous combinations of muscle activation patterns could be used to complete the whole-body task. Some methodological limitations of our muscle activity and coactivation analyses were that we did not consider effects such as muscle moment arms or contributions of deep muscles. We did explore other methods of normalization such as using the mean or maximum amplitudes of various sets of trials and also considered dividing the EMG data into functional bins such as moving out or braking. Regardless, we did not find statistically significant differences in RMS EMG or RMS coactivation between phases.

One potential implication of the stability-maneuverability tradeoff is that individuals who may self-restrict movement, such as older adults, are more likely to exhibit the tradeoff. Tasks with uncertainty could be used to promote maneuverability and to encourage these individuals to explore implicitly their movement and stability space. This exploration may help these individuals exploit the stability-maneuverability tradeoff and to identify consequently movement strategies with sufficient stability that maximize maneuverability. Learning to be less stable is not necessarily maladaptive for individuals who may over-emphasize stability.

These results demonstrate that there is a tradeoff between stability and maneuverability during a goal-directed whole-body movement. Subjects adopted movement control strategies that were more maneuverable but less stable during conditions with uncertainty. The stability-maneuverability tradeoff was not merely the consequence of inversely related behaviors or definitions in our experiment. Furthermore, our results reveal that the tradeoff manifests when stability is restricted or compromised. Individuals who self-restrict movement may benefit from training in conditions with uncertainty to learn to be more maneuverable yet sufficiently stable.

## Materials and Methods

### Ethics statement

The (University of Colorado at Boulder) Institutional Review Board has approved this protocol (0510.6) in accordance with federal regulations, university policies and ethical standards for the protection of human subjects. All subjects gave written informed consent before participation, in accordance with the University of Colorado's Institutional Review Board.

### Goal-directed whole-body movement task

Eleven subjects (age 26.2±4.7 yrs) performed goal-directed whole-body movements while standing on a narrow beam balance board. The goal-directed whole-body movement required subjects to lean or “fall” forward to shift their weight which moved a cursor (0.3 cm radius) on a computer monitor to a target (1.5 cm radius) (Figure 1). Subjects had to settle within the target for 300 ms. The baseline target distance was ∼80% of their maximum forward lean. To determine the subject's maximum anterior forward lean, we instructed subjects to stand with their feet ∼30 cm (12 in.) apart, cross their arms in front of their chest, and keep their heels in contact with the board. We then asked subjects to lean forward as far as they could and used the mean value of five maximal forward lean trials. The cursor movement was fixed to be in the center of the screen width and thus, only provided subjects with visual feedback of anterior-posterior movements. Visual feedback was also scaled on the computer screen to be twice the actual movement distances.

### Balance board

Subjects performed this forward lean, “controlled falling” task while standing on a balance board that had side-to-side (frontal plane) instability. The balance board was a flat wooden board (61.0×45.7 cm; 2×1.5 ft) with either a wide (45.7 cm) or narrow (2.5 cm) beam of support underneath the standing surface. The wide beam balance board provided subjects with ample practice to learn the anterior-posterior goal-directed movement task before completing the protocol on the narrow beam balance board. Additionally, the narrow beam balance board served to challenge frontal stability and increase muscle coactivation. By artificially increasing coactivation, we could observe if healthy young subjects could possibly learn to reduce coactivation. The beam height was 4.45 cm. The maximum tilt of the board was ∼8°, which posed minimal risk to falling but provided subjects with feedback that they had lost their balance. The balance board rested on a forceplate (AMTI LG-6-4-1). We aligned the center of the balance board with the center of the force plate. When performing the movement task, we instructed subjects to keep the balance board level, avoiding side-to-side tilts.

### Uncertainty: target-jumps

We used target uncertainty to increase maneuverability demands of the movement task. For these trials, we shifted the final target location mid-movement either backward −2 cm (jump-b), forward +2 cm (jump-f), or no shift of 0 cm (jump-0) in center of pressure coordinates along the anterior-posterior axis. When the cursor crossed 50% of the target distance, the target location shifted (target-jump). Each target-jump distance (−2, 0, or 2 cm) was presented 100 times, in a randomized order, during the uncertainty, target-jump condition block.

### Experimental protocol

The experimental protocol consisted of 200 trials to a single target location (Null), 300 target-jump trials (target-jump), and then 100 trials to the initial single target (Post) (Figure 1). Subjects first completed the protocol on a wide beam (45.7 cm) balance board for practice. Subjects were given a rest period of at least 30 seconds after every 40–50 trials and were asked to sit down during this rest period. Subjects could rest for longer periods if needed or could request additional rest periods to minimize fatigue.

### Data acquisition

We collected surface electromyography EMG data (Delsys Trigno) from sixteen lower limb muscles: tibialis anterior (TA), soleus (SO), medial gastrocnemius (MG), lateral gastrocnemius (LG), peroneus longus (PL), the long head of biceps femoris (BF), rectus femoris (RF), and vastus lateralis (VL) muscles on each lower limb. We used the SENIAM guidelines for electrode placement (http://seniam.org/). For each muscle belly surface, we shaved and cleaned the skin area with alcohol. The fixed inter-electrode distance on the Delsys Trigno sensor was 1 cm and the signal bandwidth was 20–450 Hz. We sampled EMG data at 2000 Hz. We high-pass filtered the EMG data with a fourth order zero-lag Butterworth filter at a cutoff of 20 Hz, full wave rectified the EMG, and then low-pass filtered the EMG at a cutoff of 10 Hz to get a linear envelope. We calculated the RMS EMG for the 100 ms interval before the cursor left the home circle and then averaged the last 15 successful null trials during the practice set on the wide beam board. By normalizing to this value, changes in EMG within a trial, across trials, and across beams were more easily interpreted.

We also collected forceplate data and game-related data (i.e. cursor movement, target-jump times, etc) at 200 Hz from the computer system that was devoted to the real-time virtual environment. To synchronize the forceplate data with the EMG data, we used the Delsys Trigno system trigger module and programmed the computer game to output a signal that triggered the start and stop of the Delsys system for each trial.

### Data analysis

We processed forceplate data with a fourth order low-pass Butterworth filter with zero lag (cutoff frequency = 10 Hz). We excluded trials where subjects lost their balance and allowed the balance board to tilt. These trials had medio-lateral center of pressure excursions greater than 2.8 cm, which was 1.1x the width of the narrow beam or when the medio-lateral center of pressure excursion went beyond the beam edges. Additionally, we excluded trials in which subjects took more than 2.5 seconds to reach the target. On average, 21% of null trials, 37% of target-jump trials, and 6% of post trials were excluded (i.e. failures). The remaining trials, where the board did not tilt and subjects reached the target in less than 2.5 seconds, were considered successful and included in the analysis.

### Statistical analysis

We used a Shapiro-Wilk test to check for normality of the stability margin and response time data. We used a planned comparison two-tailed paired t-test (α = 0.05) for response time (early versus late jump-b) and stability margin (late null versus late jump-0) to determine if center of pressure control strategies were significantly more maneuverable and less stable, respectively. To test for differences in RMS EMG between the late null and late jump-0 phases within a beam, we used a repeated measures analysis of variance (rmANOVA) with phase nested within beam, beam nested within side (i.e. left or right), side, and subject as a random effect for each muscle. We used the same rmANOVA structure to test for differences in RMS EMG between the early and late jump-b phases. To test for differences in RMS coactivation, we used a rmANOVA with phase nested within beam, beam, and subject as a random effect. If the rmANOVAs indicated a significant difference (*p*<0.05) for an effect, we used a Tukey-Kramer honestly significant difference (THSD) post hoc to determine differences within phases or beams (*p*<0.05). All statistical analyses were performed in JMP 9 software (SAS Institute, Inc.).

## References

[1] Jindrich DL, Qiao M (2009). Maneuvers during legged locomotion.. Chaos.

[2] Hasan Z (2005). The human motor control system's response to mechanical perturbation: Should it, can it, and does it ensure stability?. Journal of Motor Behavior.

[3] Ting LH, van Antwerp KW, Scrivens JE, McKay JL, Welch TDJ (2009). Neuromechanical tuning of nonlinear postural control dynamics.. Chaos.

[4] Full RJ, Kubow T, Schmitt J, Holmes P, Koditschek D (2002). Quantifying dynamic stability and maneuverability in legged locomotion.. Integrative and Comparative Biology.

[5] Burdet E, Osu R, Franklin DW, Milner TE, Kawato M (2001). The central nervous system stabilizes unstable dynamics by learning optimal impedance.. Nature.

[6] Hof AL, Gazendam MGJ, Sinke WE (2005). The condition for dynamic stability.. Journal of Biomechanics.

[7] Horak FB (2006). Postural orientation and equilibrium: what do we need to know about neural control of balance to prevent falls?. Age Ageing.

[8] Maki BE, McIlroy WE (1996). Postural control in the older adult.. Clinics in Geriatric Medicine.

[9] Patton JL, Lee WA, Pai YC (2000). Relative stability improves with experience in a dynamic standing task.. Experimental Brain Research.

[10] Popovic MR, Pappas IPI, Nakazawa K, Keller T, Morari M (2000). Stability criterion for controlling standing in able-bodied subjects.. Journal of Biomechanics.

[11] van Emmerik REA, van Wegen EEH (2000). On variability and stability in human movement.. Journal of Applied Biomechanics.

[12] Franklin DW, So U, Kawato M, Milner TE (2004). Impedance control balances stability with metabolically costly muscle activation.. J Neurophysiol.

[13] Bierbaum S, Peper A, Karamanidis K, Arampatzis A (2010). Adaptational responses in dynamic stability during disturbed walking in the elderly.. Journal of Biomechanics.

[14] Franklin DW, Burdet E, Osu R, Kawato M, Milner TE (2003). Functional significance of stiffness in adaptation of multijoint arm movements to stable and unstable dynamics.. Exp Brain Res.

[15] Benjuya N, Melzer I, Kaplanski J (2004). Aging-induced shifts from a reliance on sensory input to muscle cocontraction during balanced standing.. Journals of Gerontology Series a-Biological Sciences and Medical Sciences.

[16] Hortobagyi T, DeVita P (2000). Muscle pre- and coactivity during downward stepping are associated with leg stiffness in aging.. Journal of Electromyography and Kinesiology.

[17] Cenciarini M, Loughlin PJ, Sparto PJ, Redfern MS (2010). Stiffness and Damping in Postural Control Increase With Age.. Ieee Transactions on Biomedical Engineering.

[18] Liu D, Todorov E (2007). Evidence for the flexible sensorimotor strategies predicted by optimal feedback control.. Journal of Neuroscience.

[19] Duncan PW, Weiner DK, Chandler J, Studenski S (1990). FUNCTIONAL REACH - A NEW CLINICAL MEASURE OF BALANCE.. Journals of Gerontology.

[20] Feldman F, Robinovitch SN (2004). Elderly nursing home and day care participants are less likely than young adults to approach imbalance during voluntary forward reaching.. Experimental Aging Research.

[21] Feldman F, Robinovitch SN (2005). Neuromuscular versus behavioural influences on reaching performance in young and elderly women.. Gait & Posture.

[22] Seidler-Dobrin RD, He J, Stelmach GE (1998). Coactivation to reduce variability in the elderly.. Motor Control.

[23] Hortobagyi T, Solnik S, Gruber A, Rider P, Steinweg K (2009). Interaction between age and gait velocity in the amplitude and timing of antagonist muscle coactivation.. Gait & Posture.

[24] Ostrosky KM, Vanswearingen JM, Burdett RG, Gee Z (1994). A Comparison of Gait Characteristics in Young and Old Subjects.. Physical Therapy.

[25] Ketcham CJ, Seidler RD, Van Gemmert AW, Stelmach GE (2002). Age-related kinematic differences as influenced by task difficulty, target size, and movement amplitude.. J Gerontol B Psychol Sci Soc Sci.

[26] Thoroughman KA, Shadmehr R (1999). Electromyographic correlates of learning an internal model of reaching movements.. J Neurosci.

[27] Gribble PL, Mullin LI, Cothros N, Mattar A (2003). Role of cocontraction in arm movement accuracy.. Journal of Neurophysiology.

[28] van Wegen EEH, van Emmerik REA, Riccio GE (2002). Postural orientation: Age-related changes in variability and time-to-boundary.. Human Movement Science.

[29] O'Connor SM, Kuo AD (2009). Direction-Dependent Control of Balance During Walking and Standing.. Journal of Neurophysiology.

